# Learning from China’s journey to achieve malaria elimination

**DOI:** 10.1136/bmj.r758

**Published:** 2025-04-22

**Authors:** Mamadou Alpha Diallo, N Regina Rabinovich

**Affiliations:** 1 Centre International de Recherche et de Formation en Génomique Appliquée et de Surveillance Sanitaire (CIGASS), Cheikh Anta Diop University, Fann, Dakar, Senegal; 2 ISGlobal, Barcelona, Spain

## Abstract

The country’s success is a call to action for other affected areas

China, burdened with an estimated 30 million malaria cases annually in the 1940s, reached a historic milestone when the World Health Organization certified it malaria-free in 2021. This achievement—in a vast and diverse country of 1.4 billion people—offers crucial lessons for the global malaria fight, especially at a time when 94% of the 263 million cases and 95% of the 597 000 malaria deaths still occur in Africa.^1^ Achieving and sustaining control and elimination remain challenging, particularly in the current climate of funding uncertainty, and when considering the sustained effort and investment needed in China over 70 years in the face of both successes and setbacks.

Global and national funding for malaria control has led to major gains in child survival and supported progress towards elimination, with 45 countries and one territory now having achieved this milestone. In countries with ongoing transmission, WHO estimated that 177 million cases and 1million deaths were averted in 2023.^1^ Despite the challenges that remain—biological, political, and financial—25 countries reported fewer than 10 cases. However, funding has never approached more than 50% of the levels required for full implementation.

Africa has many countries heavily dependent on external donors. The US has been the largest funder, providing 37% of the $4bn available, and endemic countries provided 33%. If replenishment of the Global Fund (which supports malaria control) and the Global Alliance for Vaccines and Immunisation (funding for malaria vaccines) declines in 2025, malaria cases and deaths are predicted to increase. The hard won gains of the past two decades would be rapidly reversed.

The BMJ Collection on malaria control lessons from China explores the country’s path to elimination and how it may inform strategies elsewhere (www.bmj.com/collections/malaria-control-china).^2^ ^3^ ^4^ ^5^ ^6^ The collection examines China’s national strategy, presents case studies from Hainan and Yunnan provinces and the Huai River Basin, and details the evolving interventions that ultimately led to elimination. It offers important lessons for countries pursuing malaria control and elimination, especially in Africa, where those goals have broad political support.^7^

## International lessons

The first lesson is that comprehensive and adaptive strategies are essential. In Hainan Province, elimination was achieved through long term use of overlapping interventions—bed nets, indoor spraying, drug administration, and sustained surveillance. The approach evolved with changing risk levels, showing that persistent but flexible strategies can succeed even in highly endemic areas.

Second, success can breed complacency—and resurgence. In the Huai River Basin, early success in the 1980s led to reduced surveillance and funding, resulting in malaria resurgence in the early 2000s. Control was restored only after reintroducing mass drug administration, robust vector control, and community mobilisation. This highlights the danger of prematurely scaling back efforts and the need for long term vigilance, and echoes experiences in other countries that achieved elimination. At low levels of disease, there is the potential for lagging attention and a clear risk of resurgence.^8^

Third is the importance of cross border and cross sector coordination. Yunnan Province faced challenges from imported cases and multiple vector species along the border with Myanmar and other endemic countries. Success hinged on cross border collaboration, real time data sharing, and joint interventions. Cooperation will be vital in Africa, where national borders are often porous and populations are mobile.^8^ Collaborations with agriculture, housing, and water management sectors may also greatly reduce malaria transmission.^9^

Fourth is that larval source management deserves greater attention. China’s integration of agricultural innovations—such as rice–fish co-culture and targeted larvicide, substantially reduced mosquito breeding. Though not widely recommended by WHO, such approaches may be valuable in certain African settings. Novel tools such as drones, artificial intelligence, and remote mapping of bodies of water, merit both evaluation and policy attention.^10^

The final lesson is the importance of local data and operational research. China’s strategies were informed by surveillance data and local studies. External models are useful but empowering national programmes to generate, interpret, and act on their own data and realities is key. Sustaining surveillance as a national priority is critical and must be extended to the post-certification phase.^11^

Novel approaches to financing are urgently needed. These should be led by national governments and their ministries of finance and include regional banks and innovative partnerships. Moreover, risks from biological challenges such as drug and insecticide resistance point to the parallel need for new tools and local research.

China’s malaria-free status is a public health triumph, but it is also a call to action. Elimination requires long term tenacity; not just funds, but data driven foresight. As global malaria efforts face shifting donor priorities and resulting financing gaps, *The BMJ*’s new collection offers timely insights for policy makers, implementers, and funders alike. China and other countries that have achieved elimination have shown us the destination—now the challenge is translation to action for those left behind.

## Notes

Provenance and peer review: Commissioned; not externally peer reviewed.
